# Editorial: HPV-Associated Cancers, Socio-Economic Disparity, and Vaccination

**DOI:** 10.3389/fonc.2015.00223

**Published:** 2015-10-13

**Authors:** Ghazi Alsbeih

**Affiliations:** ^1^King Faisal Specialist Hospital & Research Centre, Riyadh, Saudi Arabia

**Keywords:** human papillomavirus, HPV prevalence, HPV genotype, cervical cancer, penile cancer, HIV–HPV coinfections, cervical cancer screening, social inequalities

I am privileged to guest edit this Frontiers Research Topic addressing HPV infection and cancer disparities with the expert assistance of Dr. Silvia De Sanjose and Dr. Ala-Eddin Al Moustafa. We are, hereby, thankful to authors of manuscripts who had responded and enriched the topic with their valuable contributions. The findings of these manuscripts are interesting and contribute to our understanding of the complexity of HPV infection, socio-economic inequalities, and related cancers in a number of unprivileged populations.

Giorgi Rossi and colleagues present an algorithm to model the impact on inequalities of introducing HPV-based screening of cervical cancer (1). They found that knowledge about HPV and cervical cancer was lower in women with low socio-economic status and in disadvantaged groups. The authors conclude that the introduction of HPV test may increase population coverage of the screening programs, but non-compliance to protocols and interaction with opportunistic screening can increase the existing inequalities.

Two papers explore HPV infection and cervical cancer screening in migrant populations. Rodriguez-Sales and colleagues examine the coverage of cervical cancer screening and prevalence of cytological abnormalities in Catalonia by immigration status (2). They found that cytology coverage was higher among immigrants who also had a higher prevalence of abnormal Pap smears compared to the Spanish-born women. The authors recommend to prioritize cervical cancer screening activities on a regular base in new comers. Tornesello and colleagues assess the prevalence and HPV genotypes among 499 immigrant women living in Southern Italy (3). They found high burden of HPV infection, lower participation in screening programs, and high incidence of cervical cancer in migrant women compared to native Italian. The authors preconize the implementation of preventive strategies to increase screening, vaccine coverage, and monitoring of uncommon HPV genotypes potentially spreading in settled population.

Wood and colleagues report on the Canadian first nations (FN) women who endure a disproportionate burden of ill health in contrast to the mainstream population (4). The authors discuss a participatory action research project, named Anishinaabek Cervical Cancer Screening Study (ACCSS), investigating the factors underlying the cervical cancer burden in FN women. ACCSS integrates community engagement, education, and culturally appropriate screening strategies to reduce the burden of cervical disease in FN communities.

Two manuscripts deal with HPV-related cancers in Middle-Eastern countries Saudi Arabia and Syria, both lacking national screening and vaccination programs for cervical cancers (5, 6). The papers presented interesting preliminary findings because there are limited studies about the prevalence of HPV in the developing countries of the Middle East. Although cervical cancer is uncommon in Saudi Arabia, the study indicates that HPV prevalence and genotypes’ distribution show similar pattern as in the world. Both studies emphasize the importance of vaccination against HPV infection. Nevertheless, a proper cost-effectiveness analysis is required to justify to health authorities the implementation of a costly vaccine. The authors call for more studies to ascertain the real prevalence of HPV at the population level at large, its association with various types of cancers, and also the impact of local tradition, emerging behavioral trends to travel for tourism or immigrations that could affect HPV transmission.

Hernandez and colleagues assessed the HPV prevalence in 79 invasive penile cancer patients diagnosed in 1998–2005 within 7 U.S. cancer registries (7). HPV DNA was present in 63% of cases with 17 viral genotypes detected. This is higher than the estimated 40–50% of penile cancers worldwide. Penile cases diagnosed in more recent years are more likely to be HPV-positive. The authors concluded that the relatively high prevalence of HPV in their study population provides evidence for a more prominent and, possibly, increasing role of HPV infection in penile carcinogenesis.

Another interesting manuscript by Vogt and colleagues deals with the concordance of oral and genital HPV infection in South Africa (8). They state that oral HPV prevalence is similar in men and women and is slightly higher in human immunodeficiency virus (HIV)-infected (13/34) subjects. Genital HPV prevalence is significantly higher than oral HPV. Oral–oral HPV concordance between couples is low, but oral–genital and genital–genital HPV concordance is higher, including concordance of male oral HPV infection with their partners’ vaginal HPV infection. The authors conclude that the data is consistent with possible transmission of vaginal HPV infection to the oral cavity of sexual partners.

Two manuscripts deal with HPV in women infected with the HIV (9, 10). McKenzie and colleagues report data on the HPV genotypes prevalent in 23 histological samples of HIV-infected women with cervical intraepithelial neoplasia in Miami, FL, USA (9). The results suggest that cervical dysplasia specimens of HIV-infected women are more likely (55%) to contain non-16 and -18 high-risk HPV types. The authors conclude that vaccine development should be more rigorously explored to ensure that this highly vulnerable population receives appropriate primary prevention. McDonald and colleagues report data on 1,371 HIV-positive women and 8,050 HIV-negative women from Cape Town, South Africa (10). In this study, HPV prevalence was higher in HIV-positive (52.4%) compared to HIV-negative women (20.8%). The authors conclude that screening strategies incorporating HPV genotyping and vaccination should be effective in preventing cervical cancer in both HIV-positive and -negative women living in sub-Saharan Africa.

This compilation of manuscripts addresses important health disparities with regard to HPV infection and related cancers. HPV infection and cervical cancer is of international interest, as almost 85% of 530,000 cases diagnosed in 2012 worldwide occurred in low- and middle-income countries. The social and economic impact of HPV infection is substantial, as cervical cancer disproportionately affects more young women. Although health authorities in many countries do not currently address these issues, it is important to emphasize that HPV-related cancers can be diagnosed early with screening and are largely preventable with vaccination.

## Conflict of Interest Statement

The author declares that the research was conducted in the absence of any commercial or financial relationships that could be construed as a potential conflict of interest.
